# Teacher evaluation as a psychological work condition: professional feelings and occupational wellbeing among teachers in Chinese private higher education

**DOI:** 10.3389/fpsyg.2026.1877720

**Published:** 2026-07-03

**Authors:** Yuda He, Jinghan Su

**Affiliations:** Azman Hashim International Business School, Universiti Teknologi Malaysia, Kuala Lumpur, Malaysia

**Keywords:** job demands-resources model, occupational wellbeing, organizational justice, private higher education, professional feelings, self-determination theory, teacher evaluation

## Abstract

**Introduction:**

Teacher evaluation can operate not only as an administrative performance-management procedure but also as a psychological work condition. This study examined whether teachers’ perceptions of evaluation were associated with occupational wellbeing through professional feelings.

**Methods:**

A cross-sectional self-report survey was conducted with 476 teachers from four private higher education institutions in Guangdong Province, China. Teacher evaluation was measured through perceptions of purpose, methods, indicators, content, feedback, and use of results. Reliability, factorability, correlations, regression models, and mediation analysis were examined in SPSS Statistics 26.0.

**Results:**

Perceived teacher evaluation was positively associated with occupational wellbeing. Evaluation content and feedback showed stronger associations with wellbeing than evaluation purpose. Professional feelings partially mediated the association between overall teacher evaluation and occupational wellbeing.

**Discussion:**

The findings suggest that evaluation systems are psychologically relevant when teachers interpret them as fair, comprehensive, feedback-rich, and recognition-oriented. The results extend organizational justice theory, self-determination theory, and the Job Demands-Resources model in a private higher education context.

## 1 Introduction

Teacher evaluation is a central performance-management practice in educational organizations. Although it is often designed to monitor teaching quality, allocate rewards, or support professional development, teachers may experience evaluation as more than a technical procedure. It can communicate whether their work is visible, whether institutional procedures are fair, and whether feedback is useful for growth (Danielson and McGreal, 2000; DeNisi and Murphy, 2017; Schleicher et al., 2018). In this sense, teacher evaluation can function as a psychological work condition rather than only an administrative mechanism.

Teacher occupational wellbeing is also a central concern in organizational psychology because teachers are employees whose motivation, commitment, and work experience are shaped by formal organizational practices. Teacher wellbeing has been associated with satisfaction, professional identity, achievement, positive affect, social relationships, and strain-related dimensions of work (Van Horn et al., 2004; Hascher and Waber, 2021; Dreer, 2023). Performance-management systems may therefore matter not only for institutional accountability but also for how employees interpret their professional value and workplace belonging (Aguinis, 2019; Aguinis and Burgi-Tian, 2021).

The present study focuses on Chinese private higher education. Private higher education is a useful setting because evaluation practices may be connected with promotion, salary adjustment, workload allocation, contract renewal, training opportunities, and professional recognition. These links make evaluation consequential for teachers’ everyday organizational experience. Guangdong Province was selected because it provides a relevant private higher education context in which institutions operate under strong pressures for quality assurance, competition, and human-resource development. Internationally, private higher education has expanded rapidly, and performance-management systems are increasingly used to govern academic work (Levy, 2006; Altbach et al., 2009). In China, recent education-evaluation reform has also emphasized the governance and developmental functions of evaluation in educational institutions (The State Council of the People's Republic of China, 2020). Therefore, the study speaks to a broader organizational psychology question: how do formal evaluation systems become psychologically meaningful for employees in high-pressure knowledge-work organizations?

Three gaps motivate the study. First, teacher evaluation has often been discussed as an administrative or quality-assurance system rather than as a perceived psychological work condition. Second, many studies examine teacher evaluation as a general practice, while fewer distinguish the potentially different psychological roles of evaluation purpose, methods, indicators, content, feedback, and use of results. Third, the psychological mechanism through which perceived evaluation is associated with occupational wellbeing remains insufficiently specified. Professional feelings—teachers’ perceived respect, recognition, belonging, competence, and confidence—may explain why the same formal evaluation system can be experienced as either supportive or pressuring.

Teacher evaluation refers to the systematic collection, interpretation, and use of information about teachers’ professional performance, teaching quality, academic work, service contribution, professional conduct, and development needs (Danielson and McGreal, 2000; Stronge and Tucker, 2003; Elliott, 2015). In higher education, it may include student evaluation, peer review, classroom observation, self-evaluation, administrative review, teaching portfolios, research assessment, and institutional service assessment. From a workplace perspective, teacher evaluation performs both administrative and developmental functions: it informs decisions about promotion, salary, rewards, contract renewal, workload, and training, while also signaling institutional expectations and defining what types of work are valued.

Organizational justice theory explains why the design and implementation of evaluation matter psychologically. Employees respond not only to outcomes but also to procedures, interpersonal treatment, and the quality of explanations they receive (Colquitt, 2001). Applied to teacher evaluation, this means that teachers may interpret evaluation as fair when criteria are transparent, evidence sources are appropriate, feedback is respectful, and result use is consistent. Conversely, opaque procedures, narrow criteria, or punitive result use may weaken trust and professional value even when the formal system appears technically complete.

Self-determination theory provides a second explanation. Wellbeing is supported when the needs for autonomy, competence, and relatedness are satisfied (Deci and Ryan, 2000; Ryan and Deci, 2017). Evaluation may support competence when feedback is specific and actionable, autonomy when teachers participate in reflection and improvement planning, and relatedness when evaluation is delivered through respectful dialogue. The work-related basic need satisfaction literature similarly shows that autonomy, competence, and relatedness are central to workplace functioning (Van den Broeck et al., 2010).

The Job Demands-Resources model further clarifies the dual role of evaluation. Evaluation can become a job demand when it increases pressure, ambiguity, insecurity, and emotional strain. It can become a job resource when it provides clarity, recognition, feedback, and developmental support (Schaufeli and Bakker, 2004; Bakker and Demerouti, 2007; Bakker and de Vries, 2021). The same formal practice may therefore have different psychological meanings depending on how teachers perceive its purpose, content, feedback, and use. This study treats teacher evaluation as a multidimensional psychological work condition rather than as a single administrative variable.

Occupational wellbeing refers to employees’ positive psychological and emotional experience in relation to work. For teachers, occupational wellbeing includes satisfaction with work, professional identity, sense of achievement, positive emotion, perceived value, interpersonal experience, and strain-related aspects of work life. This multidimensional treatment is consistent with Van Horn et al.'s (2004) occupational wellbeing model among teachers, which includes affective, cognitive, professional, social, and psychosomatic dimensions, and with recent reviews showing that teacher wellbeing is a broad construct rather than a single job-satisfaction indicator (Hascher and Waber, 2021; Dreer, 2023).

Teacher evaluation is conceptualized through six dimensions. Evaluation purpose concerns whether teachers perceive evaluation as developmental, accountability-oriented, or control-oriented. Evaluation methods refer to the sources and procedures used to collect evidence. Evaluation indicators refer to the standards used for judgment. Evaluation content refers to the work domains included in the system, such as teaching, research, student guidance, ethics, curriculum development, and institutional service. Evaluation feedback refers to the timeliness, clarity, usefulness, and developmental quality of information returned to teachers. Use of evaluation results refers to how results are applied to promotion, income, training, recognition, workload, or contract decisions. These dimensions reflect teacher-evaluation and performance-management literature that emphasizes standards, evidence sources, feedback, developmental use, and the balance between summative and formative purposes (Danielson and McGreal, 2000; Stronge and Tucker, 2003; Elliott, 2015; Ford and Hewitt, 2020).

A critical distinction exists between formal purpose and experienced procedure. A policy may state that evaluation is developmental, but teachers may judge the system through observable content, criteria, feedback, and result use. Evaluation content is important because it communicates whether teachers’ real work is visible. If student guidance, emotional labor, course coordination, curriculum development, or institutional service are excluded, teachers may experience under-recognition. Evaluation feedback is also important because it is the point where evaluation becomes an interpersonal and developmental exchange. Feedback utility has been identified as central to professional learning rather than mere reporting (Hattie and Timperley, 2007; Tuytens and Devos, 2011; Paufler et al., 2020).

Professional feelings refer to teachers’ immediate psychological interpretation of their professional value within the organization. In this study, professional feelings include perceived respect, belonging, recognition, competence, and confidence. The construct is not treated as a direct copy of one standardized scale. Rather, it is theoretically grounded in work-related basic psychological need satisfaction, organizational justice, and teacher occupational wellbeing literature (Colquitt, 2001; Van den Broeck et al., 2010; Van Horn et al., 2004). It captures how teachers interpret whether the organization recognizes their professional worth and supports their role identity.

Teacher evaluation may be associated with professional feelings in several ways. Fair procedures may be interpreted as respect. Comprehensive evaluation content may make teachers feel that their actual work is recognized. Useful feedback may strengthen competence and confidence. Transparent result use may support organizational trust and belonging. Conversely, opaque methods, narrow indicators, punitive use of results, or unhelpful feedback may weaken professional feelings. This mechanism links the three theoretical perspectives: organizational justice explains fairness and recognition, self-determination theory explains competence and relatedness, and the Job Demands-Resources model explains whether evaluation is experienced as a resource or a demand.

To address the gaps above, this study examines whether teachers’ perceptions of evaluation are associated with occupational wellbeing and whether professional feelings partially mediate this association. By integrating organizational justice theory, self-determination theory, and the Job Demands-Resources model, the study contributes to organizational psychology by explaining how evaluation systems may operate as resources or demands through teachers’ subjective interpretation of professional value. Based on this theoretical argument, the study addressed the following research questions and hypotheses:

- *RQ1*: Is perceived teacher evaluation associated with teachers’ occupational wellbeing? *H1*: Perceived teacher evaluation is positively associated with occupational wellbeing.
- *RQ2*: Do different dimensions of teacher evaluation show different strengths of association with occupational wellbeing? *H2*: Different dimensions of teacher evaluation show different strengths of association with occupational wellbeing.
- *RQ3*: Are evaluation content and evaluation feedback more strongly associated with occupational wellbeing than evaluation purpose? *H3*: Evaluation content and evaluation feedback are more strongly associated with occupational wellbeing than evaluation purpose.
- *RQ4*: Do professional feelings mediate the association between perceived teacher evaluation and occupational wellbeing? *H4*: Professional feelings partially mediate the relationship between perceived teacher evaluation and occupational wellbeing.

Additional analyses examined whether evaluation perceptions and occupational wellbeing differed by gender, age, educational level, academic title, working years, and monthly income. These demographic and professional variables were treated as contextual checks rather than as the central theoretical contribution.

## 2 Materials and methods

### 2.1 Research design and setting

This study used a quantitative cross-sectional self-report survey design. The design was appropriate because the research questions concerned current associations among perceived teacher evaluation, professional feelings, and occupational wellbeing rather than experimentally identified causal effects. A self-report approach was used because the central constructs were subjective perceptions of organizational practices and psychological work experience. The manuscript therefore interprets the findings as associations and avoids claims that evaluation reform directly causes changes in wellbeing.

The study was conducted in four private higher education institutions in Guangdong Province, China. The institutions are reported as Institution A, Institution B, Institution C, and Institution D to protect organizational confidentiality. Guangdong private higher education was selected as a relevant organizational context because private institutions often face pressure to strengthen teaching quality, staff performance, and institutional competitiveness while also retaining and motivating teachers. In this setting, teacher evaluation can have both developmental and contractual meanings for employees.

### 2.2 Participants and procedure

The target population was teachers working in private higher education institutions in Guangdong Province. A balanced quota sampling strategy was used to prevent one institution from dominating the dataset and to maintain comparable institutional representation. The intended institutional quota was approximately one quarter of the final sample from each participating institution. Within each institution, teachers were invited through institutional contact persons and departmental communication channels. The sampling was therefore quota-based and convenience-accessed rather than random, a limitation that is reported transparently because nonprobability access is often used when random access to the full population is not feasible (Etikan et al., 2016).

A total of 500 questionnaires were distributed. After incomplete or invalid responses were removed, 476 valid questionnaires were retained, producing a valid response rate of 95.2%. Each institution contributed 119 valid responses. The final sample included teachers with different genders, age groups, educational backgrounds, academic titles, working years, and income levels, as shown in Table 1.

**Table 1:** Demographic and professional profile of respondents (*N* = 476).

Variable	Category	*n*	%
Institution	Institution A	119	25.0
Institution	Institution B	119	25.0
Institution	Institution C	119	25.0
Institution	Institution D	119	25.0
Gender	Male	214	45.0
Gender	Female	262	55.0
Age	Under 36	178	37.4
Age	36–45	112	23.5
Age	46–55	142	29.8
Age	Above 55	44	9.2
Education	Bachelor or below	277	58.2
Education	Master	178	37.4
Education	Doctorate	21	4.4
Academic title	No title or assistant	155	32.6
Academic title	Lecturer	184	38.7
Academic title	Associate professor	101	21.2
Academic title	Professor	36	7.6
Working years	<3	88	18.5
Working years	3- < 5	126	26.5
Working years	5–10	164	34.5
Working years	>10	98	20.6
Monthly income	<RMB 4,000	95	20.0
Monthly income	RMB 4,000- < 6,000	178	37.4
Monthly income	RMB 6,000- < 8,000	143	30.0
Monthly income	> = RMB 8,000	60	12.6

Percentages are based on the final analytic sample of 476 teachers. Range categories are presented in ascending order.

Participation was voluntary and anonymous. Respondents were informed that the survey was used for academic research, that no personally identifiable information would be reported, and that they could decline or discontinue participation. The questionnaire did not ask respondents to provide names, identification numbers, or contact information. These procedural safeguards were used to reduce evaluation apprehension and common-method concerns in self-report research (Podsakoff et al., 2003, 2012).

The questionnaire was administered in Chinese. The English wording reported in the manuscript and supplementary materials is a conceptual translation of the Chinese questionnaire rather than a separate English-language administration. The self-report process involved reading an information statement, confirming voluntary participation, completing demographic/professional questions, and responding to items on teacher evaluation, professional feelings, and occupational wellbeing. Responses were screened for completeness and response validity before analysis. No qualitative interviews were conducted in this study.

### 2.3 Measures

Only items with ordered response categories that could be scored directionally were used to compute composite scale scores for the main analyses. Non-directional categorical items, such as the perceived main use of evaluation results or the main form of feedback, were used for contextual interpretation and were not included in composite scale scores. Supplementary Table S1, provided as a separate supplementary Word file, maps each questionnaire item to its construct, response format, scoring direction, reverse-coding status, and analytic use.

Teacher evaluation was operationalized as teachers’ perception of the fairness, clarity, comprehensiveness, usefulness, and developmental value of the evaluation system. The measurement structure was informed by teacher-evaluation and performance-management literature on formative and summative evaluation, evaluation standards, evidence sources, feedback, and result use (Danielson and McGreal, 2000; Stronge and Tucker, 2003; Elliott, 2015; DeNisi and Murphy, 2017; Schleicher et al., 2018; Ford and Hewitt, 2020).

Professional feelings were operationalized as teachers’ perceived respect, recognition, belonging, competence, and confidence in their professional role. This operationalization was grounded in organizational justice theory, self-determination theory, work-related basic need satisfaction, and teacher occupational wellbeing research (Colquitt, 2001; Deci and Ryan, 2000; Ryan and Deci, 2017; Van den Broeck et al., 2010; Van Horn et al., 2004).

Occupational wellbeing was operationalized as teachers’ positive work-related psychological experience, including satisfaction, professional identity, achievement, interpersonal experience, positive emotion, perceived value, and strain-related aspects of work life. The multidimensional treatment follows occupational wellbeing and teacher wellbeing research (Van Horn et al., 2004; Diener et al., 1999; Hascher and Waber, 2021; Dreer, 2023). Negatively worded occupational wellbeing items were reverse-coded where appropriate so that higher scores represented higher wellbeing.

Internal consistency was assessed with Cronbach’s alpha. Factorability was examined through Kaiser-Meyer-Olkin (KMO) values and Bartlett’s tests of sphericity, which are commonly used as preliminary diagnostics before interpreting scale structure in multivariate analysis (Hair et al., 2019). The teacher evaluation scale showed excellent overall reliability (alpha = 0.91), professional feelings showed excellent reliability (alpha = 0.90), and occupational wellbeing showed excellent reliability (alpha = 0.93). KMO values were 0.908 for teacher evaluation and 0.952 for occupational wellbeing, and Bartlett’s tests were significant (*p* < 0.001), supporting the suitability of the data for factor analysis (see Table 2).

**Table 2:** Measurement structure, scoring, reliability, and factorability evidence.

Construct or dimension	Item coverage and scoring	Example item content	Reliability/factorability evidence
Teacher evaluation	Ordered items covering evaluation purpose, methods, indicators, content, feedback, and use of results. Higher scores indicate more positive perceptions. Non-directional categorical items were not included in composite scores.	Reasonableness of methods; reasonableness of indicators; evaluation includes teaching, research, student guidance, professional conduct, feedback, and results use.	Overall alpha = 0.91; dimension alphas = 0.78–0.86. KMO = 0.908 and Bartlett test *p* < 0.001 supported factorability.
Professional feelings	Six 5-point Likert indicators. Higher scores indicate stronger perceived respect, recognition, belonging, competence, and confidence.	Pride in being a higher education teacher; perceived professional value; feeling respected by students; recognition from leaders.	Alpha = 0.90. Items were theoretically grounded in self-determination theory, organizational justice, and teacher wellbeing research.
Occupational wellbeing	Multidimensional 5-point Likert scale. Negatively worded strain items were reverse-coded so that higher scores indicate higher occupational wellbeing.	Professional pride, work interest, compensation satisfaction, institutional fairness, colleague relationships, workload experience, recognition, fatigue, and negative health-related items.	Alpha = 0.93. KMO = 0.95*2 and Bartlett test* p < 0.001 supported factorability.

The full item mapping is provided in Supplementary Table S1. Alpha, Cronbach’s alpha; KMO, Kaiser-Meyer-Olkin.

### 2.4 Data analysis

SPSS Statistics 26.0 (IBM Corporation, 2019) was used for the quantitative analyses. SPSS was suitable for the analyses conducted in this manuscript, including descriptive statistics, reliability analysis, factorability checks, independent-sample t tests, one-way analysis of variance, Pearson correlations, multiple regression, and regression-based mediation analysis. The significance level was set at *p* < 0.05. Effect sizes were interpreted cautiously, with correlations understood as small, moderate, or strong in relation to conventional social-science benchmarks and the study context.

The analysis strategy followed the research questions. Descriptive statistics summarized the sample and main variables. Reliability and factorability checks assessed measurement adequacy. Group comparisons were used only as contextual checks and are summarized in Supplementary Table S2. Pearson correlations examined bivariate associations among evaluation dimensions, professional feelings, and occupational wellbeing. Regression models tested whether overall teacher evaluation and the six evaluation dimensions were associated with occupational wellbeing. Tolerance and variance inflation factor values were checked to assess multicollinearity, and residual diagnostics were inspected to evaluate regression assumptions.

Mediation was estimated using a regression-based mediation procedure in SPSS consistent with PROCESS Model 4 (Hayes, 2018). Professional feelings were entered as the mediator between overall teacher evaluation and occupational wellbeing. The indirect effect was evaluated with 5,000 bootstrap samples and 95% confidence intervals. The mediation results are interpreted as indirect associations rather than evidence of causal mediation because the data were cross-sectional and self-reported. Missing or invalid questionnaires were removed before analysis; no imputation was conducted for the final analytic sample.

No qualitative coding technique, content analysis, or functional analysis was used. The only coding in the study was numerical coding of survey responses for quantitative analysis, reverse-coding of negatively worded occupational wellbeing items, and exclusion of non-directional categorical items from composite scale scores.

## 3 Results

### 3.1 Preliminary measurement evidence and descriptive statistics

Descriptive statistics showed above-midpoint perceptions of evaluation practice, professional feelings, and occupational wellbeing. Evaluation purpose received the lowest mean score, whereas evaluation content and evaluation indicators received relatively higher scores. Reliability values ranged from acceptable to excellent. The measurement evidence reported in Table 3 was therefore sufficient for the correlational, regression, and mediation analyses that followed.

**Table 3:** Descriptive statistics, reliability, and bivariate correlations.

Variable	Mean	SD	Cronbach alpha	r with professional feelings	r with occupational wellbeing
Evaluation purpose	3.18	0.82	0.78	0.18**	0.08
Evaluation methods	3.72	0.76	0.82	0.29**	0.25**
Evaluation indicators	3.83	0.70	0.84	0.31**	0.27**
Evaluation content	3.86	0.69	0.86	0.39**	0.32**
Evaluation feedback	3.79	0.73	0.85	0.36**	0.31**
Use of evaluation results	3.75	0.72	0.83	0.33**	0.29**
Overall teacher evaluation	3.69	0.55	0.91	0.41**	0.34**
Professional feelings	3.54	0.70	0.90	–	–
Occupational wellbeing	3.61	0.68	0.93	–	–

**p < 0.01. Higher values represent more positive evaluation perceptions, stronger professional feelings, or higher occupational wellbeing.

### 3.2 Answer to RQ1 and H1: overall teacher evaluation and occupational wellbeing

RQ1 asked whether perceived teacher evaluation was associated with teachers’ occupational wellbeing. The bivariate results showed that overall teacher evaluation was positively correlated with occupational wellbeing (*r* = 0.34, *p* < 0.01), and the regression model showed that overall teacher evaluation positively predicted occupational wellbeing (beta = 0.34, *p* < 0.001). The model explained 11.6% of the variance in occupational wellbeing. These results support H1. The association was moderate rather than large, indicating that teacher evaluation is one psychologically relevant work condition but not the sole determinant of wellbeing.

### 3.3 Answer to RQ2/H2 and RQ3/H3: dimension-level differences

RQ2 asked whether different dimensions of teacher evaluation showed different strengths of association with occupational wellbeing. The bivariate correlations and multiple regression model indicated that the dimensions were not equally associated with wellbeing. Evaluation content and evaluation feedback showed the strongest associations, while evaluation purpose was weak and non-significant when the other dimensions were included. These results support H2.

RQ3 asked whether evaluation content and evaluation feedback were more strongly associated with occupational wellbeing than evaluation purpose. In the multiple regression model, evaluation content (beta = 0.22, *p* < 0.001) and evaluation feedback (beta = 0.18, *p* < 0.001) had the strongest standardized coefficients. Evaluation purpose was not significant (beta = 0.03, *p* = 0.542). These results support H3 and suggest that teachers’ wellbeing is more closely linked to what evaluation recognizes and what feedback provides than to the declared purpose of evaluation alone. VIF values were below 2.00, indicating that multicollinearity was not a serious concern (see Table 4).

**Table 4:** Regression models predicting occupational wellbeing.

Model/predictor	Standardized beta	*t*	*p*	Tolerance	VIF
Model 1: overall teacher evaluation	0.34	7.88	< 0.001	1.000	1.000
Model 2: evaluation purpose	0.03	0.61	0.542	0.780	1.282
Model 2: evaluation methods	0.12	2.41	0.016	0.655	1.527
Model 2: evaluation indicators	0.10	1.98	0.049	0.618	1.618
Model 2: evaluation content	0.22	4.30	< 0.001	0.550	1.818
Model 2: evaluation feedback	0.18	3.56	< 0.001	0.592	1.689
Model 2: use of evaluation results	0.14	2.76	0.006	0.633	1.580

Model 1: *R*^2^ = 0.116, adjusted *R*^2^ = 0.114, *F*(1, 474) = 62.09, *p* < 0.001. Model 2: *R*^2^ = 0.166, adjusted *R*^2^ = 0.156, *F*(6, 469) = 15.57, *p* < 0.001.

### 3.4 Answer to RQ4 and H4: professional feelings as mediator

RQ4 asked whether professional feelings mediated the association between perceived teacher evaluation and occupational wellbeing. Teacher evaluation was positively associated with professional feelings, and professional feelings were positively associated with occupational wellbeing. The total association between teacher evaluation and occupational wellbeing was significant. When professional feelings were included as the mediator, the direct association remained significant but was reduced. The bootstrap confidence interval for the indirect effect did not include zero, indicating a significant indirect association through professional feelings. These results support H4 and are consistent with partial mediation. Because the data are cross-sectional, the mediation result is interpreted as an indirect association rather than evidence of causal mediation (see Table 5).

**Table 5:** Mediation analysis with professional feelings as mediator.

Path	Estimate	SE	*t*	*p*	95% CI/interpretation
Teacher evaluation - > Professional feelings	0.41	0.041	9.98	< 0.001	Significant positive association
Professional feelings - > Occupational wellbeing	0.50	0.039	12.82	< 0.001	Significant positive association
Teacher evaluation - > Occupational wellbeing, total effect	0.34	0.043	7.88	< 0.001	Significant total association
Teacher evaluation - > Occupational wellbeing, direct effect	0.13	0.041	3.17	0.002	Reduced but significant direct association
Indirect effect through professional feelings	0.21	–	–	< 0.001	95% bootstrap CI [0.15, 0.28]

The indirect-effect confidence interval was estimated with 5,000 bootstrap samples. Estimates are interpreted as associations because the design was cross-sectional.

### 3.5 Additional contextual checks and conceptual interpretation

Additional demographic and professional group comparisons were conducted as contextual checks and are reported in Supplementary Table S2. These analyses suggested that age, academic title, working years, and monthly income were associated with some differences in evaluation perceptions or occupational wellbeing, whereas gender and educational level showed no meaningful difference in the reported models. These checks are not treated as the central theoretical contribution because the main purpose of the study was to examine perceived evaluation, professional feelings, and wellbeing.

**Figure 1:**
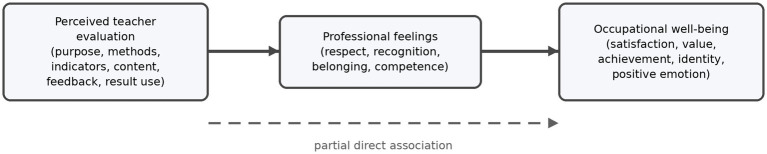
Conceptual model of teacher evaluation, professional feelings, and occupational wellbeing.

Figure 1 summarizes the conceptual interpretation of the findings. Perceived teacher evaluation is treated as a multidimensional psychological work condition. It is associated with occupational wellbeing both directly and indirectly through professional feelings. The partial mediation result suggests that formal evaluation systems become psychologically meaningful when teachers interpret them as signals of respect, recognition, belonging, and competence.

## 4 Discussion

### 4.1 Principal findings and comparison with previous studies

This study examined how teachers’ perceptions of evaluation were associated with occupational wellbeing in Chinese private higher education. Four findings are central. First, perceived teacher evaluation was positively but moderately associated with occupational wellbeing, supporting H1 while indicating that evaluation is one component of a broader work experience. Second, evaluation dimensions were not equally associated with wellbeing. Evaluation content and feedback showed stronger associations than evaluation purpose, supporting H2 and H3. Third, professional feelings partially mediated the association between teacher evaluation and occupational wellbeing, supporting H4. Fourth, the findings were consistent with the theoretical argument that evaluation becomes psychologically relevant through teachers’ interpretation of fairness, need support, recognition, and work-resource value.

The positive association between perceived teacher evaluation and occupational wellbeing is broadly consistent with performance-management research showing that appraisal systems influence employee attitudes not only through outcomes but also through perceived fairness, feedback quality, and developmental value (DeNisi and Murphy, 2017; Schleicher et al., 2018; Aguinis, 2019; Aguinis and Burgi-Tian, 2021). The present study extends this literature by examining teachers in Chinese private higher education as organizational employees rather than treating evaluation only as an educational quality-assurance issue.

The stronger role of evaluation content and feedback is similar to teacher-evaluation and feedback research emphasizing that professional learning depends on meaningful standards, useful feedback, and formative use of evaluation information (Hattie and Timperley, 2007; Tuytens and Devos, 2011; Ford and Hewitt, 2020; Paufler et al., 2020). At the same time, the present findings differ from work that emphasizes stated evaluation purpose alone: in this sample, declared purpose was weak once concrete design dimensions were considered. This difference suggests that teachers may evaluate the psychological meaning of appraisal systems through observable content, feedback, and result use rather than through official purpose statements only.

The partial mediation through professional feelings is consistent with organizational justice theory and self-determination theory because fair procedures, respectful treatment, competence support, and relatedness are central to positive workplace functioning (Colquitt, 2001; Deci and Ryan, 2000; Van den Broeck et al., 2010; Ryan and Deci, 2017). It is also compatible with the Job Demands-Resources model, in which evaluation may function as a job resource when it provides clarity, recognition, and developmental support, but as a demand when it creates ambiguity or insecurity (Schaufeli and Bakker, 2004; Bakker and Demerouti, 2007; Bakker and de Vries, 2021).

The moderate size of the associations is theoretically important because it prevents the overstatement that evaluation alone determines occupational wellbeing. Teachers’ wellbeing is also likely to be connected with workload, income, leadership, collegial relationships, professional development, job security, and personal circumstances. Nevertheless, evaluation remains relevant because it communicates what the institution values and whether teachers’ work is recognized.

### 4.2 Integration with the three theoretical perspectives

From an organizational justice perspective, the results suggest that evaluation content and feedback are not merely technical components of appraisal. They are signals of procedural and interactional justice. When evaluation content includes the real scope of teachers’ work, teachers may perceive that their contributions are visible. When feedback is useful and respectful, teachers may perceive that the organization treats them as professionals rather than as objects of control. This interpretation extends justice theory by showing that the visibility of work can be a fairness issue in teacher evaluation (Colquitt, 2001; DeNisi and Murphy, 2017).

From a self-determination theory perspective, evaluation feedback and developmental use may be associated with wellbeing because they are connected to competence, autonomy, and relatedness. Feedback can support competence by clarifying how teachers can improve. Participatory reflection and improvement planning can support autonomy. Respectful dialogue can support relatedness by showing that the institution recognizes teachers’ professional identity. The mediation through professional feelings is consistent with this need-supportive interpretation (Deci and Ryan, 2000; Van den Broeck et al., 2010; Ryan and Deci, 2017).

From the Job Demands-Resources perspective, the study shows why evaluation can have mixed meanings. Evaluation may be experienced as a demand when it emphasizes surveillance, narrow indicators, pressure, or high-stakes consequences. It may be experienced as a resource when it provides clarity, recognition, feedback, and developmental support. The findings therefore suggest that the psychological role of evaluation depends less on the mere presence of an evaluation system and more on how teachers perceive its content, feedback, and result use (Schaufeli and Bakker, 2004; Bakker and Demerouti, 2007).

### 4.3 Why evaluation content and feedback mattered more than purpose

One of the clearest findings is that evaluation content and feedback were more strongly associated with occupational wellbeing than evaluation purpose. This does not mean that purpose is irrelevant. Rather, it suggests that declared purpose must be translated into observable procedures. Teachers do not experience evaluation mainly through official statements. They experience it through what work domains are recognized, what criteria are used, what feedback they receive, and how results are applied.

Evaluation content matters because teachers’ work is multidimensional. In private higher education institutions, teachers may prepare lessons, deliver classes, guide students, revise curricula, coach competitions, communicate with students and families, undertake research tasks, and perform institutional service. When evaluation content is narrow, important work may become invisible. When evaluation content is comprehensive, evaluation can support recognition and procedural fairness because it acknowledges the real scope of teachers’ work. This interpretation is consistent with teacher-evaluation literature that emphasizes the importance of broad standards and multiple forms of evidence (Danielson and McGreal, 2000; Stronge and Tucker, 2003; Elliott, 2015).

Feedback matters because it is the point at which evaluation becomes a psychological interaction. A score or ranking alone tells teachers that they have been judged, but it does not necessarily support growth. Developmental feedback can support competence by clarifying improvement pathways, relatedness by enabling respectful dialogue, and autonomy by helping teachers participate in professional-development planning. This explanation is consistent with feedback research showing that feedback is effective when it is specific, interpretable, and linked to improvement (Hattie and Timperley, 2007; Tuytens and Devos, 2011; Paufler et al., 2020).

### 4.4 Practical implications

The practical implications should be read as design considerations based on associative evidence, not as proof that specific reforms will cause wellbeing improvement. First, institutions should review whether their evaluation content captures the actual scope of teachers’ work. Where teachers are expected to provide student guidance, curriculum development, mentoring, institutional service, or professional conduct, these responsibilities should not be invisible in evaluation. Second, institutions should strengthen feedback quality. Feedback should be timely, specific, respectful, and connected to training, mentoring, or improvement plans. Third, institutions should make result use transparent. When the same evaluation process is linked to growth, salary, promotion, and contract renewal, teachers may question whether developmental language conceals control. Clear rules and differentiated use of results may reduce this concern.

These implications are especially relevant to private higher education because evaluation often carries both developmental and contractual consequences. A transparent and feedback-rich evaluation system may support professional feelings, whereas a narrow or punitive system may increase compliance while weakening long-term motivation and occupational wellbeing.

### 4.5 Limitations and future research

Several limitations should be noted. First, the data were collected from four private higher education institutions in Guangdong Province, so the findings may not generalize to public universities, vocational colleges, or private institutions in other regions. Future research should test the model in other institutional types and national contexts. Second, the cross-sectional design prevents strong causal conclusions. Longitudinal, time-lagged, or intervention-based studies are needed to examine whether changes in evaluation practices are followed by changes in professional feelings and wellbeing.

Third, all substantive variables were measured by self-report questionnaires, which may involve common method bias (Podsakoff et al., 2003, 2012). The study reduced this concern by using anonymous responses, separating constructs conceptually, and interpreting results cautiously, but future research should use multi-source data, administrative records, supervisor feedback, or qualitative interviews. Fourth, construct overlap is possible because professional feelings and occupational wellbeing both include positive psychological experiences. Future studies should use confirmatory factor analysis and more differentiated measurement models to test discriminant validity.

Fifth, the model did not include all possible predictors of occupational wellbeing. Workload, leadership style, organizational climate, income satisfaction, job security, professional-development opportunities, psychological safety, work engagement, burnout, and personal resilience may also be relevant. Future research could integrate these variables within the Job Demands-Resources framework and test whether teacher evaluation operates as a resource, a demand, or both. Finally, qualitative interviews or organizational ethnography could complement the survey findings by showing how teachers experience evaluation conversations, feedback meetings, and result use in everyday organizational life.

## 5 Conclusion

This study positions teacher evaluation as a psychological work condition in Chinese private higher education. Perceived teacher evaluation was positively but moderately associated with occupational wellbeing, and evaluation content and feedback were the most relevant dimensions. Professional feelings partially mediated this association, suggesting that evaluation becomes psychologically meaningful when teachers interpret it as recognition, respect, belonging, competence, and confidence. The findings contribute to organizational psychology by connecting teacher evaluation with organizational justice, self-determination theory, and the Job Demands-Resources model. Because the study used cross-sectional self-report data, the results should be interpreted as associations rather than causal effects.

## Data Availability

The raw data supporting the conclusions of this article will be made available by the authors, without undue reservation.
