# Supplementary material for: Teacher evaluation as a psychological work condition: professional feelings and occupational wellbeing among teachers in Chinese private higher education
Source: Front Psychol. 2026 Jul 3;17:1877720. doi: 10.3389/fpsyg.2026.1877720 (PMC13377977; doi:10.3389/fpsyg.2026.1877720)
Supplement: Supplementary file 1 [file Table_1.DOCX]

**Supplementary material**

This file contains supplementary tables. It does not repeat the article title, author list, affiliations, or correspondence details to avoid discrepancies between the article and supplementary material.

*Supplementary Table S1. Questionnaire item mapping and analytic use*

Note. English wording is a conceptual translation of the Chinese questionnaire. Items with non-directional categorical responses were used only for contextual interpretation and were not included in composite scale scores. Negatively worded occupational well-being items were reverse-coded where appropriate.

| **Construct** | **Dimension/domain** | **Item code** | **English item meaning** | **Response format** | **Analytic use** |
| --- | --- | --- | --- | --- | --- |
| Demographic/professional variables | Background control/context | D1 | Gender | Male; female | Contextual/group-comparison variable |
| Demographic/professional variables | Background control/context | D2 | Age group | Under 36; 36-45; 46-55; above 55 | Contextual/group-comparison variable |
| Demographic/professional variables | Background control/context | D3 | Educational level | Junior college; bachelor; master; doctorate | Contextual/group-comparison variable |
| Demographic/professional variables | Background control/context | D4 | Academic title | None/teaching assistant; lecturer; associate professor; professor | Contextual/group-comparison variable |
| Demographic/professional variables | Background control/context | D5 | Teaching experience | <3 years; 3-<5 years; 5-10 years; >10 years | Contextual/group-comparison variable |
| Demographic/professional variables | Background control/context | D6 | Monthly income | <RMB 4,000; RMB 4,000-<6,000; RMB 6,000-<8,000; >=RMB 8,000 | Contextual/group-comparison variable |
| Teacher evaluation | Evaluation purpose | T1 | Clarity of teacher-evaluation content | 4-point ordered response | Composite scale |
| Teacher evaluation | Evaluation purpose | Purpose item | Perceived institutional purpose of teacher evaluation | Nominal categorical response | Contextual only |
| Teacher evaluation | Evaluation methods | T2 | Perceived reasonableness of evaluation methods | 4-point ordered response | Composite scale |
| Teacher evaluation | Evaluation methods | T3 | Satisfaction with evaluation methods | 4-point ordered response | Composite scale |
| Teacher evaluation | Evaluation indicators | T4 | Reasonableness of evaluation cycle | 4-point ordered response | Composite scale |
| Teacher evaluation | Evaluation indicators | T5 | Reasonableness of evaluation indicators | 4-point ordered response | Composite scale |
| Teacher evaluation | Evaluation indicators | T6 | Reasonableness of indicator weights | 4-point ordered response | Composite scale |
| Teacher evaluation | Evaluation content | T13 | Evaluation includes appearance, language, and conduct requirements | 4-point ordered response | Composite scale |
| Teacher evaluation | Evaluation content | T14 | Evaluation examines dedication to work | 4-point ordered response | Composite scale |
| Teacher evaluation | Evaluation content | T15 | Evaluation examines attendance | 4-point ordered response | Composite scale |
| Teacher evaluation | Evaluation content | T16 | Evaluation emphasizes team awareness | 4-point ordered response | Composite scale |
| Teacher evaluation | Evaluation content | T17 | Evaluation examines lesson-plan preparation | 4-point ordered response | Composite scale |
| Teacher evaluation | Evaluation content | T18 | Evaluation examines standardized teaching language | 4-point ordered response | Composite scale |
| Teacher evaluation | Evaluation content | T19 | Evaluation examines use of scientific teaching methods | 4-point ordered response | Composite scale |
| Teacher evaluation | Evaluation content | T20 | Evaluation examines classroom blackboard or board design | 4-point ordered response | Composite scale |
| Teacher evaluation | Evaluation content | T21 | Student achievement is used as an important teacher-evaluation indicator | 4-point ordered response | Composite scale |
| Teacher evaluation | Evaluation content | T22 | Research outputs and published papers are used as evaluation indicators | 4-point ordered response | Composite scale |
| Teacher evaluation | Evaluation content | T23 | Number of published papers is used as an evaluation indicator | 4-point ordered response | Composite scale |
| Teacher evaluation | Evaluation content | T24 | Class-management ability is evaluated | 4-point ordered response | Composite scale |
| Teacher evaluation | Evaluation content | T25 | Students' moral education is evaluated | 4-point ordered response | Composite scale |
| Teacher evaluation | Evaluation content | T26 | Teacher attitude toward students is evaluated | 4-point ordered response | Composite scale |
| Teacher evaluation | Evaluation content | T27-content | Ability to teach according to individual student needs is evaluated | 4-point ordered response | Composite scale |
| Teacher evaluation | Evaluation content | T28-content | Communication with parents is evaluated | 4-point ordered response | Composite scale |
| Teacher evaluation | Evaluation content | T29-content | Communication with leaders is evaluated | 4-point ordered response | Composite scale |
| Teacher evaluation | Evaluation content | T30-content | Communication with colleagues is evaluated | 4-point ordered response | Composite scale |
| Teacher evaluation | Evaluation content | T31-content | Reflection on teaching behavior is evaluated | 4-point ordered response | Composite scale |
| Teacher evaluation | Evaluation content | T32-content | Active self-improvement ability is evaluated | 4-point ordered response | Composite scale |
| Teacher evaluation | Evaluation feedback | T27-feedback | Satisfaction with fairness of evaluation feedback | 4-point ordered response | Composite scale |
| Teacher evaluation | Evaluation feedback | T28-feedback | Satisfaction with evaluation feedback results | 4-point ordered response | Composite scale |
| Teacher evaluation | Evaluation feedback | T34 | Main form of evaluation feedback | Nominal categorical response | Contextual only |
| Teacher evaluation | Use of evaluation results | Result-use item | Main application of evaluation results | Nominal categorical response | Contextual only |
| Teacher evaluation | Use of evaluation results | T29-result | Whether evaluation should serve as a basis for personnel decisions | Binary response | Directional/contextual indicator |
| Teacher evaluation | Use of evaluation results | T30-result | Whether current evaluation has led the teacher to adjust work focus | Binary response | Directional/contextual indicator |
| Teacher evaluation | Use of evaluation results | T31-result | Whether current evaluation helps improve professional ability | Binary response | Directional/contextual indicator |
| Teacher evaluation | Use of evaluation results | Difference-reason item | Perceived main reason for differences in evaluation results | Nominal categorical response | Contextual only |
| Teacher evaluation | Use of evaluation results | T32-result | Perceived obviousness of differences in evaluation results among teachers | Binary response | Contextual only |
| Professional feelings | Perceived professional value/respect/competence | Q1 | Pride in being a higher education teacher | 5-point Likert response | Mediator indicator |
| Professional feelings | Perceived professional value/respect/competence | Q2 | Perceived personal value in higher education work | 5-point Likert response | Mediator indicator |
| Professional feelings | Perceived professional value/respect/competence | Q6 | Work enables realization of ambition and ideals | 5-point Likert response | Mediator indicator |
| Professional feelings | Perceived professional value/respect/competence | Q13 | Ability to complete planned teaching tasks successfully | 5-point Likert response | Mediator indicator |
| Professional feelings | Perceived professional value/respect/competence | Q15 | Feeling respected by students | 5-point Likert response | Mediator indicator |
| Professional feelings | Perceived professional value/respect/competence | Q18 | Recognition and affirmation from leaders | 5-point Likert response | Mediator indicator |
| Occupational well-being | Multidimensional occupational well-being | Q1-Q3 | Professional pride, value, and interest in teaching | 5-point Likert response | Outcome scale / reverse-coded where indicated |
| Occupational well-being | Multidimensional occupational well-being | Q4-Q6 | Stability, compensation satisfaction, and self-realization | 5-point Likert response | Outcome scale / reverse-coded where indicated |
| Occupational well-being | Multidimensional occupational well-being | Q7-Q9 | Leadership concern, decision participation, and institutional fairness | 5-point Likert response | Outcome scale / reverse-coded where indicated |
| Occupational well-being | Multidimensional occupational well-being | Q10-Q11 | Colleague relationship and interpersonal harmony | 5-point Likert response | Outcome scale / reverse-coded where indicated |
| Occupational well-being | Multidimensional occupational well-being | Q12-Q14 | Workload, task completion, and input-output satisfaction | 5-point Likert response | Outcome scale / reverse-coded where indicated |
| Occupational well-being | Multidimensional occupational well-being | Q15-Q16 | Student respect and institutional concern for teacher psychology | 5-point Likert response | Outcome scale / reverse-coded where indicated |
| Occupational well-being | Multidimensional occupational well-being | Q17 | Stress from research and title evaluation (reverse-coded) | 5-point Likert response | Outcome scale / reverse-coded where indicated |
| Occupational well-being | Multidimensional occupational well-being | Q18-Q22 | Recognition, differentiated institutional concern, social status, holiday satisfaction, and family support | 5-point Likert response | Outcome scale / reverse-coded where indicated |
| Occupational well-being | Multidimensional occupational well-being | Q23-Q26 | Physical and emotional strain, monotony, exhaustion, and retirement intention (reverse-coded) | 5-point Likert response | Outcome scale / reverse-coded where indicated |
| Occupational well-being | Multidimensional occupational well-being | Q27 | Availability of cultural and leisure activities | 5-point Likert response | Outcome scale / reverse-coded where indicated |
| Occupational well-being | Multidimensional occupational well-being | Q28-Q31 | Collapse feeling, fatigue, boredom, and negative health effects (reverse-coded) | 5-point Likert response | Outcome scale / reverse-coded where indicated |

*Supplementary Table S2. Summary of group differences in teacher evaluation and occupational well-being*

Note. Group comparisons are reported as contextual analyses and are not treated as the central theoretical contribution of the manuscript.

| **Background variable** | **Teacher evaluation** | **Occupational well-being** | **Interpretation** |
| --- | --- | --- | --- |
| Gender | t = 0.84, p = .400 | t = 0.67, p = .505 | No meaningful difference |
| Age | F = 3.18, p = .024 | F = 3.02, p = .030 | Partial difference |
| Educational level | F = 1.75, p = .156 | F = 1.92, p = .126 | Not significant |
| Academic title | F = 5.64, p < .001 | F = 6.21, p < .001 | Clear professional-stage difference |
| Working years | F = 4.36, p = .005 | F = 4.08, p = .007 | Career-experience difference |
| Monthly income | F = 3.91, p = .009 | F = 3.54, p = .015 | Income-related difference |
